# Interaction with Ribosomal Proteins Accompanies Stress Induction of the Anticancer Metallodrug BOLD‐100/KP1339 in the Endoplasmic Reticulum

**DOI:** 10.1002/anie.202015962

**Published:** 2021-02-01

**Authors:** Benjamin Neuditschko, Anton A. Legin, Dina Baier, Arno Schintlmeister, Siegfried Reipert, Michael Wagner, Bernhard K. Keppler, Walter Berger, Samuel M. Meier‐Menches, Christopher Gerner

**Affiliations:** ^1^ Institute of Inorganic Chemistry Faculty of Chemistry University of Vienna Waehringer Str. 42 1090 Vienna Austria; ^2^ Department of Analytical Chemistry Faculty of Chemistry University of Vienna Waehringer Str. 38 1090 Vienna Austria; ^3^ Research Network “Chemistry, Microbiology and Environmental Systems Science” University of Vienna Währinger Str. 42 1090 Vienna Austria; ^4^ Institute of Cancer Research and Comprehensive Cancer Center Department of Medicine I Medical University of Vienna Borschkegasse 8a 1090 Vienna Austria; ^5^ Research Cluster “Translational Cancer Therapy Research” University of Vienna Waehringer Str. 42 1090 Vienna Austria; ^6^ Large-Instrument Facility for Environmental and Isotope Mass Spectrometry Centre for Microbiology and Environmental Systems Science University of Vienna Althanstr. 14 1090 Vienna Austria; ^7^ Core Facility Cell Imaging and Ultrastructure Research Althanstr. 14 1090 Vienna Austria; ^8^ Joint Metabolome Facility University of Vienna and Medical University of Vienna Waehringer Str. 38 1090 Vienna Austria

**Keywords:** bioinorganic chemistry, metals in medicine, multi-omics, ribosome, ruthenium

## Abstract

The ruthenium‐based anticancer agent BOLD‐100/KP1339 has shown promising results in several in vitro and in vivo tumour models as well as in early clinical trials. However, its mode of action remains to be fully elucidated. Recent evidence identified stress induction in the endoplasmic reticulum (ER) and concomitant down‐modulation of HSPA5 (GRP78) as key drug effects. By exploiting the naturally formed adduct between BOLD‐100 and human serum albumin as an immobilization strategy, we were able to perform target‐profiling experiments that revealed the ribosomal proteins RPL10, RPL24, and the transcription factor GTF2I as potential interactors of this ruthenium(III) anticancer agent. Integrating these findings with proteomic profiling and transcriptomic experiments supported ribosomal disturbance and concomitant induction of ER stress. The formation of polyribosomes and ER swelling of treated cancer cells revealed by TEM validated this finding. Thus, the direct interaction of BOLD‐100 with ribosomal proteins seems to accompany ER stress‐induction and modulation of GRP78 in cancer cells.

## Graphical abstract

Multi‐omics analysis, including affinity proteomics, revealed that the anticancer drug candidate BOLD‐100/KP1339 may target the ribosomal proteins RPL10/RPL24 and GTF2I. Swelling of the endoplasmic reticulum (ER) and the observation of polyribosomes using imaging techniques indicate that BOLD‐100 treatment may not only inhibit proliferation, but also lead to a functional disturbance of the ER.

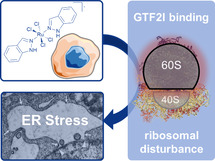

The ruthenium complex sodium *trans*‐[tetrachlorido‐bis(1*H*‐indazole)ruthenate(III)] (BOLD‐100/KP1339, Figure 1) is among the most widely investigated non‐platinum metal‐based anticancer drugs.^[1]^ It has shown promising anticancer effects in an autochthonous tumour model^[2]^ and its safety has been proven in clinical trials^[3]^ with clinical phase 1b trials under way (*NCT04421820*). In contrast to the clinically widely administered platinum‐based anticancer agents that target DNA, this ruthenium(III) anticancer drug candidate acts on other biomolecules.^[4]^ Recently, the down‐modulation of the glucose‐regulated protein of 78 kDa (GRP78, HSPA5) combined with endoplasmic reticulum (ER) stress, as well as the induction of reactive oxygen species (ROS) emerged as accepted drug effects in several 2D and 3D tumour models.^[5]^ However, the mechanism of ER‐stress induction and possible targets of BOLD‐100 remained rather elusive despite intense research efforts.

**Figure 1:**
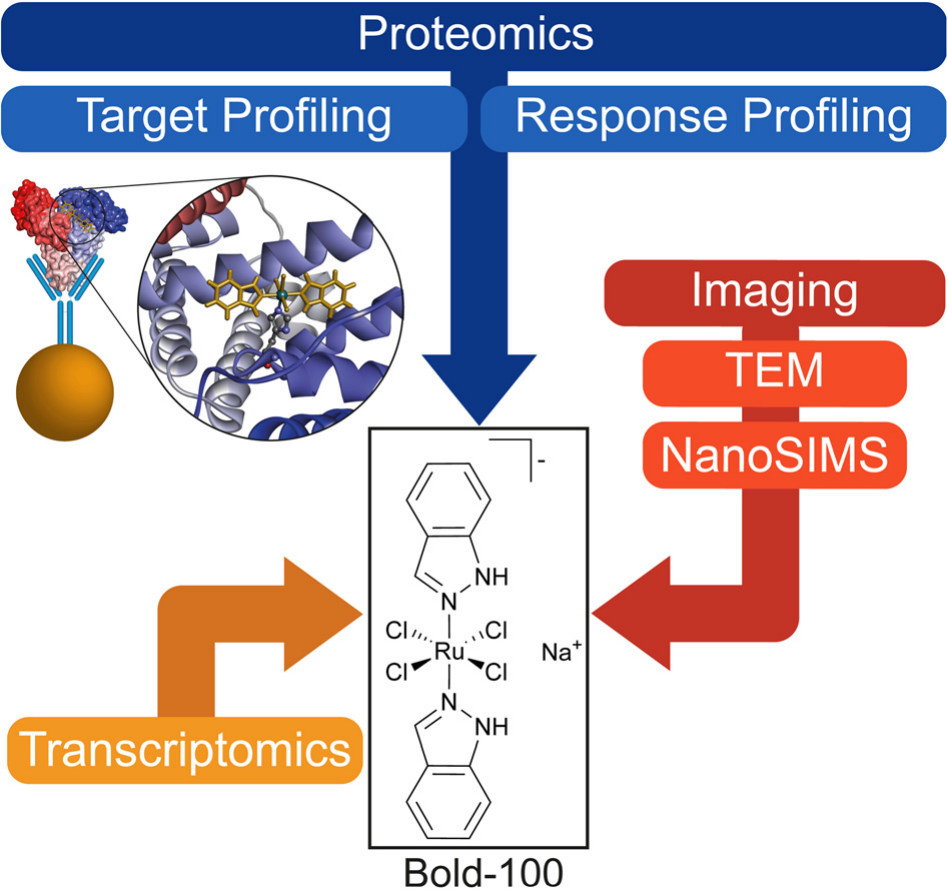
Workflow of the multi‐omics approach followed in this study. Immobilisation of BOLD‐100 with human serum albumin (HSA) and anti‐HSA antibody on magnetic beads for target profiling. This is integrated with findings from proteome profiling and global transcriptome profiling. Correlative TEM/NanoSIMS imaging was applied for ultrastructure characterization and visualization of the sub‐cellular drug distribution in HCT116 cancer cells.

Metalloproteomics is an emerging tool to elucidate potential targets and mechanisms of action for metallodrugs, and also in conjunction with other omics techniques.[^1b^, ^6^] Recently, solutions were found to identify and validate protein targets of largely substitution‐inert metal‐based anticancer agents based on gold^[7]^ and platinum,^[8]^ but also of labile organoruthenium prodrugs.^[9]^ Moreover, metalloproteomes were characterized following a similar strategy in living cells.^[10]^ Furthermore, integrating metalloproteomics with other omics layers (e.g. transcriptomics) promises to reveal unprecedented details regarding the mode of action of metal‐based anticancer agents.^[6]^

BOLD‐100 is a double‐prodrug that undergoes hydrolysis via ligand exchange of chlorido ligands and subsequent reduction to ruthenium(II).[^1a^, ^4^] The oxidation state +III was found to be relatively stable even in tissues over prolonged time periods.^[11]^ The drug features a rapid binding to human serum albumin (HSA) via non‐covalent bonding, which is transformed into a stable Ru‐HSA adduct, characterized by coordinative bonding.^[12]^ X‐ray diffraction studies further showed that the *trans*‐indazole ligands are retained in some cases,^[13]^ but may be exchanged in others.^[14]^ The natural reactivity of BOLD‐100 towards HSA is used as a tumour targeting mechanism.^[1a]^ The formation of a stable BOLD‐100‐HSA adduct was exploited here as an immobilization strategy to enable proteomic target profile experiments since this compound was not amenable for suitable derivatization so far. Here, we show that BOLD‐100 may directly interact with ribosomal proteins and provide evidence of the proposed mechanism by a multi‐omics approach in conjunction with imaging techniques based on ultrastructure‐resolving microscopy and nano‐probe secondary ion mass spectrometry (NanoSIMS).^[15]^

The study of the potential molecular targets of BOLD‐100 was carried out by adapting a previously described shotgun proteomics approach based on label‐free quantification (LFQ).^[16]^ This target‐response profiling combines the investigation of potential interacting proteins of an immobilized drug with a comprehensive perturbation study. First, HSA was immobilized on magnetic beads with anti‐HSA antibodies and then reacted with BOLD‐100 to generate the BOLD‐100‐HSA adducts (s. experimental section, Figure 1). This immobilization reflects the *in vivo* situation of the anticancer drug candidate, which rapidly binds to serum proteins within a short period of time, as shown in mouse experiments amongst others.^[17]^ Target profiling was performed using native whole cell lysates of the colon carcinoma cell line HCT116. The cell lysates were exposed to the beads for 2 h and after thorough washing the bound proteins were eluted under mild conditions with citrate buffer and then processed for analysis by shotgun proteomics. The pull‐downs were performed in a differential approach to remove non‐selective binding events. Next to a normal pull‐down experiment, a competitive pull‐down was performed by pre‐treating the cell lysate with free BOLD‐100 before exposure to the immobilized drug on the beads. This latter strategy saturates selective binding sites so that the resulting target profile includes only non‐selective binding partners. Subtracting these from the target profile obtained by normal pull‐down cancels the non‐selective interactors and provides a list of the selective binding partners.

The enrichment plot highlights the protein target profile of BOLD‐100 (Figure 2 A). The selectivity of binding is expressed as the fold‐change of enrichment between normal and competitive pull‐down experiments on the *y*‐axis. The *x*‐axis features the concomitant significance of enrichment and is expressed as a p‐value. The pull‐down experiment employing BOLD‐100‐HSA adducts provided 57 proteins as probable interaction partners, which are shown as circles in the enrichment plot, whose size is dependent on their LFQ intensity (Figure 2 A). This number is smaller compared to previously investigated metallodrugs, which may be caused by the employed HSA‐drug adducts in this study. Interestingly, proteins of the large ribosomal subunit (RPL10, RPL17, RPL23, RPL24) represent a major cluster of the target profile and suggest a direct interaction of BOLD‐100 with ribosomal constituents (Figure S1). Moreover, the protein with the highest enrichment factor corresponded to the general transcription factor II‐I (GTF2I), a transcription factor regulating the response to ER stress, for example through binding to the promoter region of HSPA5 (GRP78).^[18]^ The disruption of the promotion function of GTF2I through interaction with BOLD‐100 may therefore directly account for the previously observed down‐modulation of GRP78. Thus, the target spectrum of BOLD‐100 clearly shows potential interactors that are involved in ER homeostasis.

**Figure 2:**
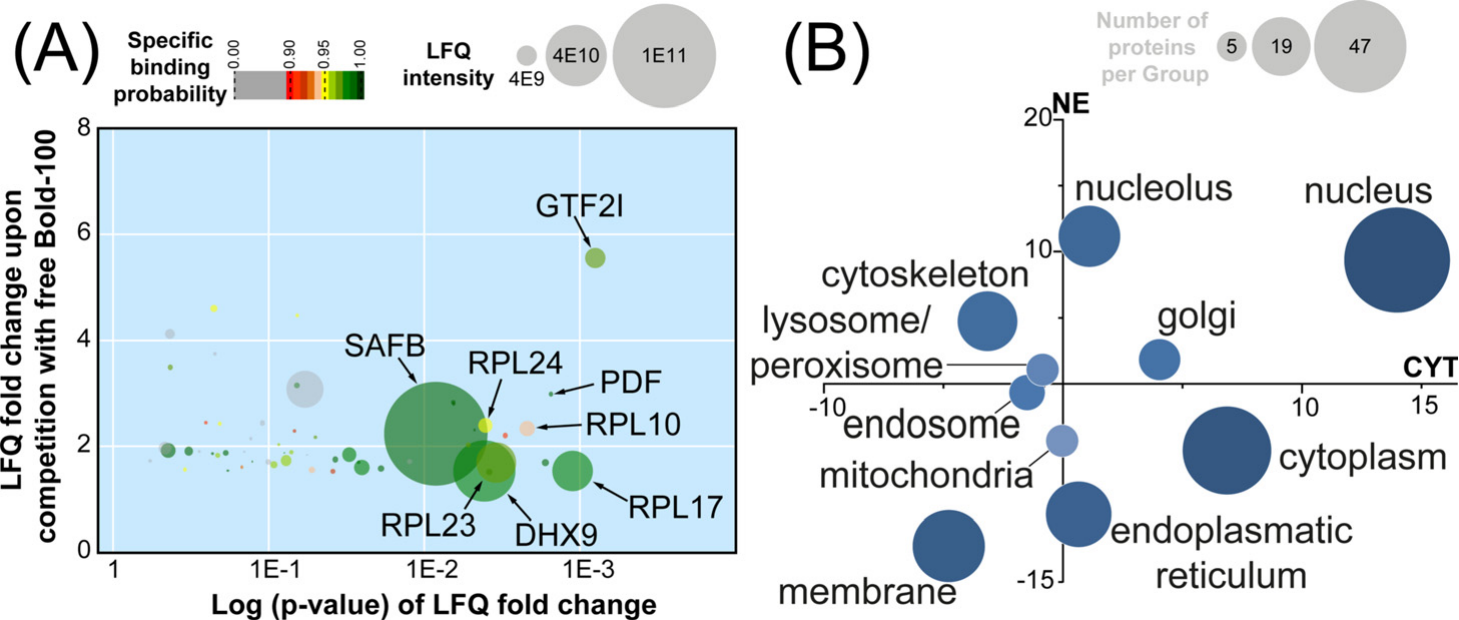
(**A**) Enrichment plot displaying potential interactors of BOLD‐100 immobilized via HSA‐adduct formation from HCT116 cell lysates. The *y*‐axis represents the LFQ fold‐change between normal and competitive pull‐downs and the associated p‐values are given on the *x*‐axis. The colour indicates the specific binding probability and the size correlates to the abundance of each protein. (**B**) Significantly regulated proteins upon treating HCT116 cancer cells with BOLD‐100 (80 μM, 24 h) were grouped according to cellular compartments and displayed according to the summed fold‐change in cytoplasmic (CYT, *x*‐axis) and nuclear fraction (NE, *y*‐axis). Circle size is determined by number of proteins in the respective group.

A response profiling experiment^[19]^ was then conducted to complement the data obtained from the pull‐down. HCT116 cancer cells were treated with BOLD‐100 (80 μM) for 24 h. The cytoplasmic (CYT) and nuclear (NE) fractions of the cells were isolated and proteolytically digested for analysis by LFQ shotgun proteomics. At this dose, the treatment featured relatively minor proteome alterations. Of a total of 4193 proteins detected applying a false‐discovery rate (FDR) of 0.01 on the peptide and protein level, 116 and 86 proteins featured significant abundance changes in CYT and NE, respectively (FDR=0.05 and S0=0.1). Thus, BOLD‐100 does not seem to affect the proteome to the extent of other ruthenium drug candidates.[^16^, ^20^] The cellular response profile is visualized as global protein expression changes to the perturbation with BOLD‐100. This is constructed by grouping the significantly regulated proteins according to their cellular compartments and subsequently plotting the summed protein fold‐changes along the CYT and NE axes (Figure 2 B). The circle size corresponds to the number of proteins in each group. A pronounced down‐regulation of ER‐associated proteins was observed in the NE fraction, while proteins associated with the nucleus and nucleolus were upregulated. In contrast to other ruthenium drug candidates, BOLD‐100 treatment did only induce minor regulations in mitochondrial proteins.^[20]^

The selection of potential interactors from the target profile and the significantly regulated proteins from the response profiling experiments were then integrated in a network using STRING^[21]^ analysis in Cytoscape^[22]^ (Figure 3 A, Figure S2). This network reveals whether potential targets are mechanistically connected to drug‐induced perturbations in the cancer cells. The obtained network showed proteins involved in three major biological processes, namely the endoplasmic reticulum unfolded protein response (blue), ribosome biogenesis (orange) and mRNA processing (red). The potential interactors are shown in green. Interestingly, the network displayed central ribosomal proteins that connect to protein regulations in ribosome biogenesis and mRNA processing. Furthermore, the analysis also showed the mentioned connection between GTF2I and HSPA5, which extended to the cluster of ER stress. Thus, the target‐response network indicated a direct interaction of BOLD‐100 with ribosomal proteins and potentially GTF2I that mediate the observed alterations in proteins of ribosome biogenesis and ER homeostasis in treated HCT116 cells.

**Figure 3:**
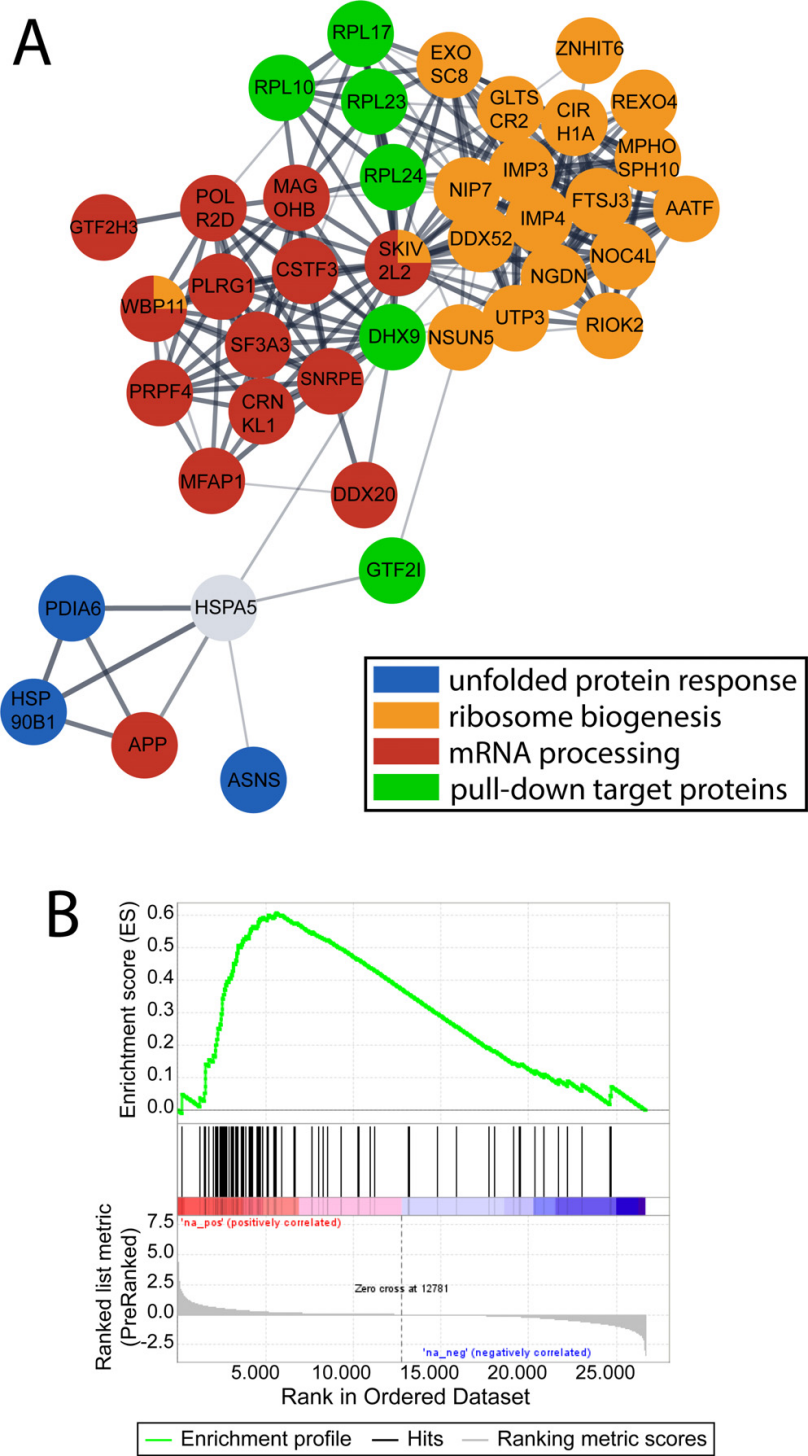
(**A**) Excerpt of the STRING analysis from the significantly regulated proteins (FDR=0.05, S0=0.1) created in Cytoscape. Colours indicate the affiliation to Gene ontology terms assigned by the STRING analysis. Blue: endoplasmic reticulum unfolded protein response (GO.0030968); Orange: ribosome biogenesis (GO.0042254); Red: mRNA processing (GO.0006397); Green: Proteins obtained in the pull‐down experiment, additional HSPA5 as known response of the drug and link to the UPR. (**B**) Gene set enrichment analysis according to the KEGG database demonstrating significant enrichment of the term “ribosome”.

The involvement of ribosomal proteins was further investigated on the mRNA level by a global transcriptome profiling. For this purpose, HCT116 cells were treated with BOLD‐100 (100 μM) for 6 h and total mRNA was isolated and analysed. Importantly, the term “ribosome”, containing multiple mRNA for ribosomal proteins, was generally up‐regulated and showed an overall enrichment score of 0.6, representing one of the most enriched gene sets ranked top 16 (Figure 3 B). mRNA of PRL10, RPL17, RPL23 and RPL24 was detected, as identified in the protein target profile, but only mRNA of RPL10 and RPL24 was upregulated.

Further support of a direct interaction of BOLD‐100 with ribosomes was previously reported by analysing Ru in treated HCT116 cell lysates using size‐exclusion chromatography coupled to inductively‐coupled plasma MS. In these studies, ruthenium was predominantly detected in the very high molecular weight fraction (>700 kDa) suggesting that this compound interacts with very large protein complexes such as ribosomes.^[23]^

Then, NanoSIMS was employed to reveal the subcellular distribution of ruthenium in HCT116 cells after treatment with BOLD‐100 for 24 h (Figure 4). The cell pellets obtained from mono‐layer grown cancer cells were embedded in a low viscosity resin and cut in slices of 100 nm thickness. Ruthenium was found homogenously distributed between major cellular compartments and no significant difference in accumulation between cytoplasm, nucleus or nucleolus was observed. The cytoplasm displayed distinct organellar hotspots with a higher tendency of ruthenium accumulation. Consequently, the largely homogenous distribution of ruthenium in the cellular interior provided further support of the direct interaction of BOLD‐100 with cytoplasmic ribosomes (Figure 4 A).

**Figure 4:**
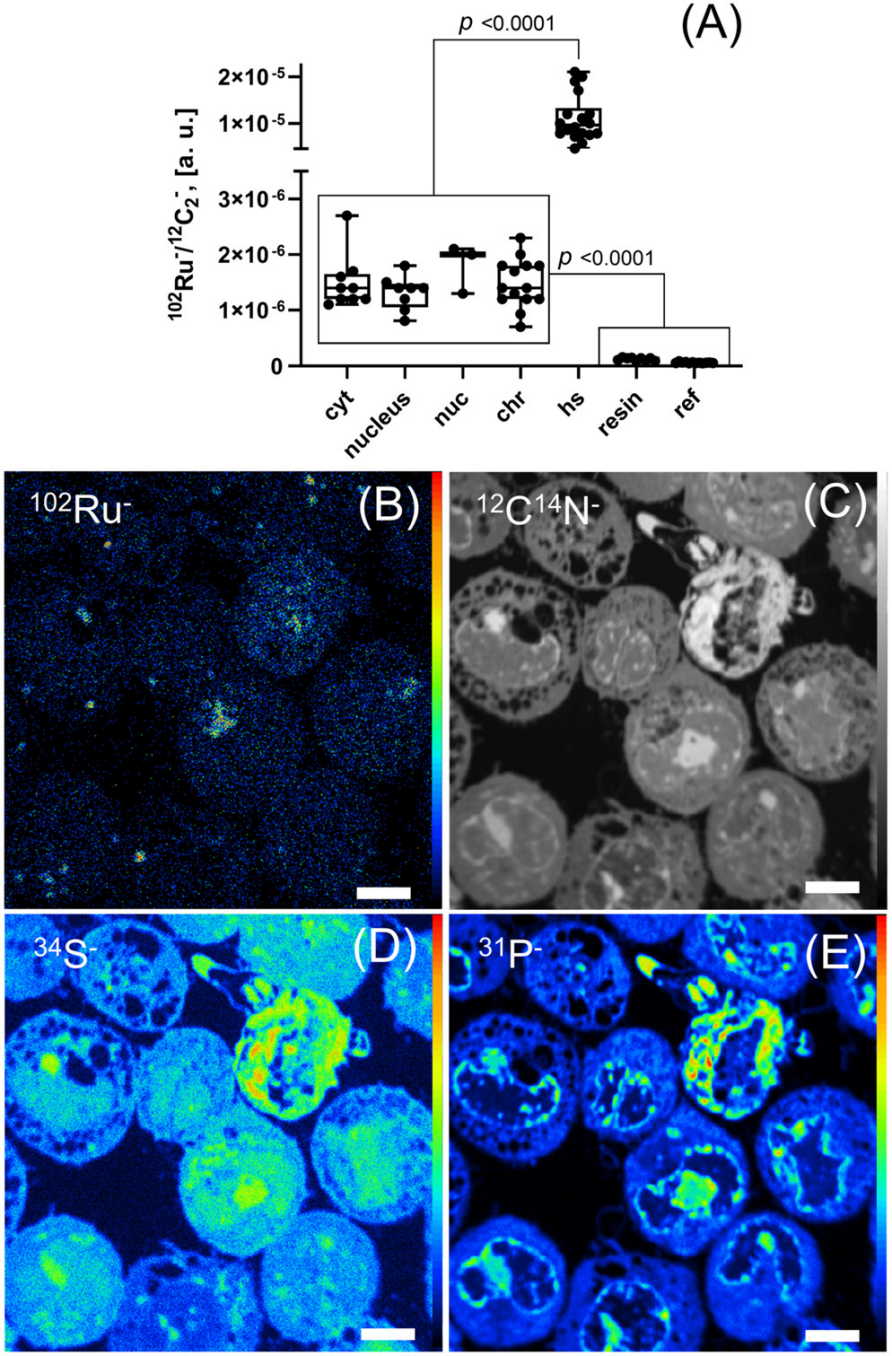
Visualization of the ruthenium distribution in BOLD‐100 treated colon carcinoma cells by means of NanoSIMS. (**A**) Evaluation of the relative ruthenium content in subcellular compartments of HCT116 cells upon exposure to BOLD‐100 (400 μM, 24 h) via region of interest (ROI) specific evaluation of the ^12^C_2_^−^ normalized ^102^Ru^−^ signal intensities. ^102^Ru^−^ (**B**), ^12^C^14^N^−^ (**C**), ^34^S^−^ (**D**) and ^31^P^−^ (**E**) secondary ion maps revealing sites of drug accumulation. Abbreviations: cyt—cytoplasm, nuc—nucleolus, chr—heterochromatin structures, hs—cytoplasmic hotspots of Ru accumulation, resin—extracellular regions (epoxy resin), ref—values from an untreated control sample. Scale bars: 5 μm.

The consecutive 100 nm section of BOLD‐100‐treated HCT116 cells embedded in resin was analysed by transmission electron microscopy (TEM) after post‐staining with gadolinium acetate and lead citrate (Figure 5). The images revealed swelling of the ER, resulting in perinuclear space (PS) exaggeration (Figure 5 A). These effects were absent in untreated controls (Figure S3). Furthermore, ribosomes were prone to detach from the ER and formed clusters of polyribosomes, which were observed as string‐like formations in the cytoplasm (Figure 5 D, asterisks). BOLD‐100 was previously found to be able to cross‐link proteins, which might underline this finding.^[24]^ Rounded single‐membrane cytoplasmic organelles with lamellar and vesicular content were observed. These vesicles overlapped with the NanoSIMS visualized cytoplasmic ruthenium hotspots and might constitute a cellular attempt of metal detoxification (Figure S4).

**Figure 5:**
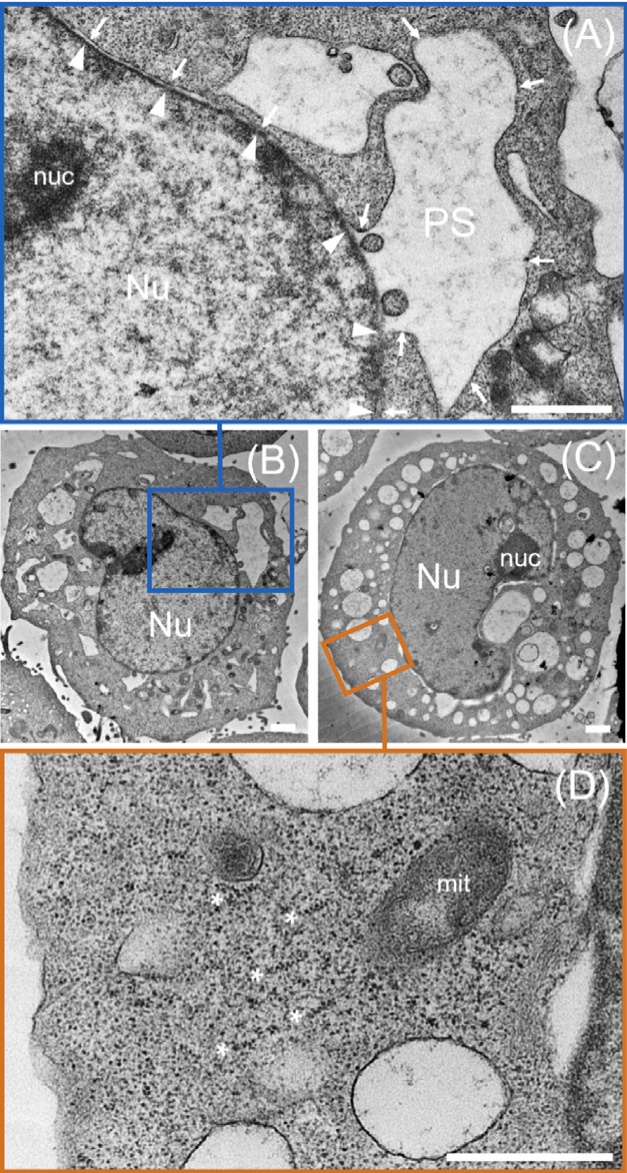
Endoplasmic reticulum stress morphology upon exposure of HCT116 cancer cells to BOLD‐100 (400 μM, 24 h). Transmission electron micrographs showing endoplasmic reticulum (ER) swelling, which results in perinuclear space (PS) exaggeration and ER vacuolization (**A**–**D**). In (A, a close‐up of B) the outer nuclear membrane continuous with ER is marked with arrows, while the inner nuclear membrane is marked with arrowheads. The ribosomes are forming clusters of polyribosomes (marked with asterisks) visible in cytoplasm (D, a close‐up of C). Abbreviations: Nu—nucleus, nuc—nucleolus, mit—mitochondria, PS—perinuclear space. Scale bars: 1 μm.

In summary, using a multi‐omics approach complemented by NanoSIMS and TEM as imaging techniques, this work highlights molecular events that could lead to the known ER stress response induced by BOLD‐100. ER stress was previously identified as a rather unique mechanism of action of this ruthenium(III) anticancer agent.[^1a^, ^1c^] BOLD‐100 seems to directly interact with ribosomal proteins, most probably with RPL10 and/or RPL24, not only resulting in the inhibition of proliferation, but also in a functional disturbance of the ER. This was further evidenced by the formation of polyribosomes, concomitant ER swelling and vesicle formation in cancer cells upon treatment with BOLD‐100 at higher concentrations.

## Conflict of interest

The authors declare no conflict of interest.

## Supporting information

As a service to our authors and readers, this journal provides supporting information supplied by the authors. Such materials are peer reviewed and may be re‐organized for online delivery, but are not copy‐edited or typeset. Technical support issues arising from supporting information (other than missing files) should be addressed to the authors.

Supplementary
