# Supplementary material for: Interaction with Ribosomal Proteins Accompanies Stress Induction of the Anticancer Metallodrug BOLD‐100/KP1339 in the Endoplasmic Reticulum
Source: Angew Chem Int Ed Engl. 2021 Feb 1;60(10):5063–8. doi: 10.1002/anie.202015962 (PMC7986094; doi:10.1002/anie.202015962)
Supplement: Supplementary file 1 — Supplementary [file ANIE-60-5063-s001.pdf]

Angewandte Chemie
Eine Zeitschrift der Gesellschaft Deutscher Chemiker

Supporting Information

# Interaction with Ribosomal Proteins Accompanies Stress Induction of the Anticancer Metallodrug BOLD-100/KP1339 in the Endoplasmic Reticulum

*Benjamin Neuditschko, Anton A. Legin, Dina Baier, Arno Schintlmeister, Siegfried Reipert, Michael Wagner, Bernhard K. Keppler, Walter Berger, Samuel M. Meier-Menches,* and Christopher Gerner**

anie_202015962_sm_miscellaneous_information.pdf

# Materials and Methods

## Compound

BOLD-100/KP1339 was synthesized as a brown powder according to published procedures.[1] BOLD-100 was always freshly dissolved in a dimethylsulfoxide (20 mM) stock solution, which was further diluted to the appropriate concentration with complete medium.

## Cell culture

HCT116 cells were kindly provided by M. Jakupec (University of Vienna). They were cultured in McCoy's 5A modified medium (Gibco, Thermo Fischer Scientific, Life Technologies, with L-glutamine) supplemented with 1% penicillin/streptomycin (Sigma-Aldrich, Austria) and 10% heat-inactivated fetal calf serum (FCS, Sigma, Austria) at 37 °C and 5% $CO_2$.

## Proteomics

**Target profiling.** HCT116 cells were grown in T75 polystyrene cell culture flasks with cell growth surface for adherent cells (Sarstedt, Austria) in three biological replicates per condition to approximately 95% confluence. The cells were washed, lysed and homogenised by passing through a gauge syringe 20-times as described elsewhere.[2] In brief, a non-denaturing lysis buffer was used for pull-down experiments consisting of NaCl (100 mM), Tris HCl (50 mM), $MgCl_2$ (1.5 mM), NaF (25 mM), NP-40 (0.2%), glycerol (5%), TLCK (10 µg $mL^{-1}$), DTT (1 mM), $Na_3VO_4$ (1 mM), PMSF (1 mM) and pepstatin/leupeptin/aprotinin (each 1 µg $mL^{-1}$), at pH 7.4. The samples were centrifuged (3500 rpm, 5 min, 4 °C) and the supernatant was used for further processing. For the competitive pull-down experiments the cell lysates were incubated with BOLD-100 (50 µM) for 1 h at 4 °C prior to exposure to the immobilised drug.

To immobilise BOLD-100 a complex with human serum albumin (HSA) was formed. Therefore, anti-HSA beads (1 mL) used for depletion of human serum (PureProteome™ Albumin Magnetic Beads, Millipore, Germany) were incubated with HSA (2 mg, 30,3 nmol, Albumin from human serum, Sigma-Aldrich, Austria) for 1 h at room temperature. After washing with PBS, BOLD-100 (95 µg, 189 nmol)

was added and incubated for 2 h at room temperature. The formed BOLD-100–HSA adduct consisting of magnetic beads, HSA, and BOLD-100 (depicted in Figure 1) was then washed and split into six aliquots used for the pulldown experiment. The cell lysates (with and without pre-incubated drug) were added to the magnetic beads and shaken for 4 h at 4 °C. Afterwards, the supernatant was removed and the beads were extensively washed with lysis buffer and HEPES buffer (HEPES-NaOH (50 mM), EDTA (0.5 mM), PMSF (1 mM), TLCK (10 µg $mL^{-1}$) and pepstatin/leupeptin/aprotinin (each 1 µg $mL^{-1}$)] at pH 8. The proteins were eluted with a citrate buffer (0.1 M, pH 2.4) and proteolytically digested using trypsin/Lys-C for LC-MS/MS analysis.

**Response profiling.** HCT116 cells ($5 \cdot 10^6$ cells) were seeded in T25 polystyrene cell culture flasks with cell growth surface for adherent cells (Sarstedt, Austria) in three biological replicates per condition and allowed to grow for 24 h. Cells of three T25 flasks were treated with BOLD-100 (80 µM) for 24 hours, while the others served as controls (including solvent control). The cells were lysed and separated into cytoplasmic and nuclear fraction as previously described.[2] In short, the cells were washed with PBS and 1 ml of isotonic lysis buffer containing protease inhibitors was added. The cells were scraped and homogenised using a gauge syringe. After centrifugation the supernatant was precipitated in ethanol overnight and the remaining nuclear pellet was swelled in hypertonic buffer and ultimately lysed with NP-40 containing buffer. The samples were again centrifuged and the supernatant precipitated in ethanol.

**Protein sample preparation.** The proteins were proteolytically digested in-solution using a filter-assisted protein digestion (FASP) protocol as described previously.[3] In short, the precipitated proteins were centrifuged at 4536 g for 30 min, the supernatant was decanted and the protein pellet dried under vacuum. After dissolving in sample buffer (7.5 M Urea, 7.5 M Thiourea, 0.1 M DTT, 4% CHAPS, 0.25% SDS)  the protein concentration was determined and 20 µg total protein per samples was used for further processing. After reduction with DTT and alkylation with iodacetamide (both Sigma-Aldrich, Austria) the proteins were digested with typsin/Lys-C (MS grade; Promega Corporation, Madison, WI, USA) and dried in a SpeedVac.

**Proteome analysis by LC-MS/MS.** For the HPLC-MS/MS analysis the peptides were resolved in 5 µL formic acid (aq. 30%) and diluted with 40 µL mobile phase A (97.9% $H_2O$, 2% acetonitrile, 0.1% formic acid). The injection volumes were 5 µL for the pull-down samples and 10 µL for samples from cytoplasmic and nuclear fractions. Chromatographic separation was carried out with a Dionex UltiMate 3000 RSLCnano LC system, which was coupled to the QExactive Orbitrap mass spectrometer (all Thermo Fisher Scientific, Austria). Peptides were trapped on a C18 2 cm × 100 µm pre-column and LC separation was performed on a 50 cm × 75 µm Pepmap100 analytical column (both Thermo Fisher Scientific, Austria). The total run time of the HPLC method for analyzing pull-down samples was 85 min and included a 43 min gradient from 7% to 40% mobile phase B (79.9% acetonitrile, 20% $H_2O$, 0.1% formic acid). For samples of the cytoplasmic and nuclear fractions were separated using a HPLC method of 135 min total run time, including a 95 min gradient from 8% to 40% mobile phase B. The flow rate was always 300 nL $min^{-1}$. Mass spectrometric settings were the same for all fractions. The resolution on the MS1 level was set to 70,000 (at *m/z* = 200) with a scan range from *m/z* 400–1,400. The top eight abundant peptide ions were chosen for fragmentation at 30% normalized collision energy and resulting fragments analyzed in the Orbitrap at a resolution of 17,500 (at *m/z* = 200).

**Proteomics Data Analysis.** Raw data were subjected to the freely available software MaxQuant (version 1.6.0.1) utilizing the Andromeda search engine, followed by statistical evaluation with the Perseus software (version 1.6.0.2). For the MaxQuant search, a minimum of two peptide identifications, at least one of them being a unique peptide, was required for valid protein identification. Digestion mode was set to "Specific" choosing Trypsin/P. The peptide mass tolerance was set to 50 ppm for the first search and to 25 ppm for the main search. The false discovery rate (FDR) was set to 0.01 both on peptide and protein level, based on the q-value. The database applied for the search was the human Uniprot database (version 03/2018, reviewed entries only) with 20,316 protein entries. Further settings for the search included carbamidomethylation as fixed modification and oxidation of methionine and acetylation of the protein C terminus as variable modifications. Each peptide was allowed to have a maximum of two missed cleavages and two modifications, "Match between runs" was enabled and the

alignment window set to 10 min, with the match time window of 1 min. Gene ontology (GO) term analysis was performed with DAVID[4] against the human gene background. The confidence of enrichment of a term is expressed by a Q-value, which is a multiple testing-corrected P-value according to Benjamini-Hochberg. The enrichment plot contained four dimensions. The y-axis represents the enrichment of a protein and is calculated as the ratio of the LFQ-areas of a given protein (three biological and two technical replicates) in the normal pull-down with respect to the competitive pull-down. The x-axis denotes the confidence of the enrichment and is obtained by a p-value of the mentioned LFQ-areas. The size of the bubbles represents the intensity in the mass spectrometric analysis. Finally, the colour represents the specific binding probability (P) and is a measure of specificity of a given protein to bind to the small molecule probe. It is obtained via the CRAPome database[5] and is calculated as follows: P = 1 –(CRAPome N° of samples containing the protein)/(CRAPome total N° of samples)·(CRAPome average SC)/(Average SC of Pull Down), where SC denotes spectralcounts. The total number (N°) of samples in the CRAPome database was 411.

Cytoscape[6] (v 3.8.0) was used for the STRING[7] analysis with a confidence score cutoff of 0,4 and 0 additional interactors. The significantly regulated proteins (FDR = 0.05, S0 = 0.1) from cytoplasm (CYT) and nuclear extract (NE) were used for the analysis. To study the enriched gene ontology terms and for the assignment of the proteins to GO terms of biological processes the ClueGO app[8] (v2.5.6) was used. The mass spectrometry proteomics data has been deposited to the ProteomeXchange Consortium via the PRIDE partner repository with the dataset identifier PXD022449.

**Total-RNA isolation and whole genome gene expression array**

Whole genome gene expression array analysis was performed as described elsewhere[9]. In brief, mRNA was isolated using the RNeasy Mini Kit (QIAGEN GmbH, Hilden, Germany) from HCT116 cells that were either untreated or treated with 100 µM of BOLD-100 for 6 h. Whole genome gene expression analyses were performed on 4 × 44K oligonucleotide microarrays (G4845A, Agilent Technologies, Santa Clara, CA, USA) according to manufacturer's recommendation. Feature extraction was carried out using the Feature Extraction software (version 11.5.1.1.). Differentially expressed genes in BOLD-

100-treated vs. untreated HCT116 cells were analysed using the GeneSpring software (version 13.0) and ranked according to their significance level in pre-defined gene sets using the Gene Set Enrichment Analysis software[10].

**Cell culture and growth conditions for TEM and NanoSIMS analysis**

The human cancer cell line HCT116 (colon carcinoma) was grown in 75 $cm^2$ culture flasks (Starlab) as adherent monolayer cultures in complete medium [i.e., RPMI 1640 supplemented with 10% fetal bovine (Biowest) and 4 mM L-glutamine (Merck)]. The cell line was officially authenticated and confirmed to be mycoplasma and cross-contamination free (Multiplexion, Germany, 2014). Cell cultures were incubated at 37 °C in a moist atmosphere containing 5% $CO_2$.

**TEM analysis and sample preparation**

HCT116 cells were harvested from culture flasks by trypsinization and seeded in 5 mm dishes (CytoOne, Starlab) in densities of $2\cdot10^6$ cells per well. Cells were allowed to grow for 72 h to reach 80-90% confluence. After growing the cells were treated with BOLD-100 solution in complete RPMI medium prepared from fresh DMSO stock (100 mM). The cells were incubated with compound for 24 h. After the treatment the cells were detached by trypsinization, washed with PBS (2×), subdivided into 1 ml aliquots (each comprising around $2.5\cdot10^6$ cells), pelleted by centrifugation (300 g), and fixed in 2.5% glutaraldehyde in 0.1 M sodium cacodylate buffer (pH 7.4, RT). After chemical fixation, cell cultures were washed with cacodylate buffer (3×), and stained with 1% $OsO_4$ (dissolved in 0.1 M sodium cacodylate buffer overnight) for 1 h. After washing the pellets with cacodylate buffer (3×) the samples were dehydrated in an ascending ethanol series (30%, 50%, 70%, 90% and 100% ethanol, 10 min each). Ethanol (100%) was then replaced with propylen oxide (2× for 5 min, 1× for 10 min). The samples were infiltrated and embedded in low viscosity resin (Agar Scientific). The resin blocks were polymerized in an oven at 60 °C for 32 h. To obtain two consecutive thin sections (100 nm in thickness, 1× for TEM, 1× for NanoSIMS) the resin-embedded cell monolayers were cut by using an ultramicrotome (Ultracut S; Leica) with a diamond knife (Diatome). The first section was placed on a copper grid, counterstained with gadolinium acetate and lead citrate, and imaged in a TEM Libra 120 (Zeiss) at 120 kV. Images

were acquired by using a bottom stage digital camera, TRS (4 MP), and iTEM software (Soft Imaging System GmbH). The second consecutive section was deposited onto antimony-doped silicon wafer platelets (7.1 ×7.1 × 0.75 mm; Active Business Company, GmbH, Germany) for spatially correlated metal distribution analysis by NanoSIMS.

**NanoSIMS analysis, image processing and numerical data evaluation**

NanoSIMS measurements were carried out on a Cameca NS 50L instrument (France), as described previously.[11] Briefly, the detectors of the multicollection assembly were positioned to enable parallel detection of $^{12}C_2^-$, $^{12}C^{14}N^-$, $^{31}P^-$, $^{34}S^-$ and $^{102}Ru^-$ secondary ions for drug distribution measurements. Prior to data acquisition, analysis areas were pre-conditioned *in situ* by rastering of a high intensity, defocused $Cs^+$ ion beam in the following sequence of high and extreme low ion impact energies (HE / 16 keV and EXLIE / 50 eV, respectively): HE at 100 pA beam current to a fluence of 5,0E14 ions/cm$^2$; EXLIE at 400 pA beam current to a fluence of 5,0E16 ions/cm$^2$; HE to a fluence of 3,1E14 ions/cm$^2$. An area of 40 × 40 µm$^2$ was scanned with 512 × 512 pixel image resolution and approx. 80 nm physical resolution (probe size). The primary ion beam was scanned with a per-pixel dwell time of 10 ms per cycle. In total, 30 cycles were acquired in each measurement. NanoSIMS image data were evaluated by using the WinImage software package (version 2.0.8) provided by Cameca. Prior to stack accumulation, the individual images were drift corrected manually. Secondary ion signal intensities were corrected for detector dead time on a per-pixel basis and quasi-simultaneous arrival (QSA) of $C_2^-$ and $CN^-$ secondary ions on a per-region of interest (ROI) basis.[12] ROI specific drug accumulation patterns were inferred from signal intensity ratios associated with Ru and the carbon matrix ($^{102}Ru^-$ and $^{12}C_2^-$, respectively) displayed in arbitrary units (a. u.). ROI data were analyzed for normal distribution (Kolmogorov-Smirnov test, $p<0.05$). Normally distributed data were tested for significant differences in the arithmetic means by application of Welch's t-test. Statistical calculations were conducted with the GraphPad Prism software. TEM/NanoSIMS correlation images were generated utilizing the GIMP software (version 2.10).

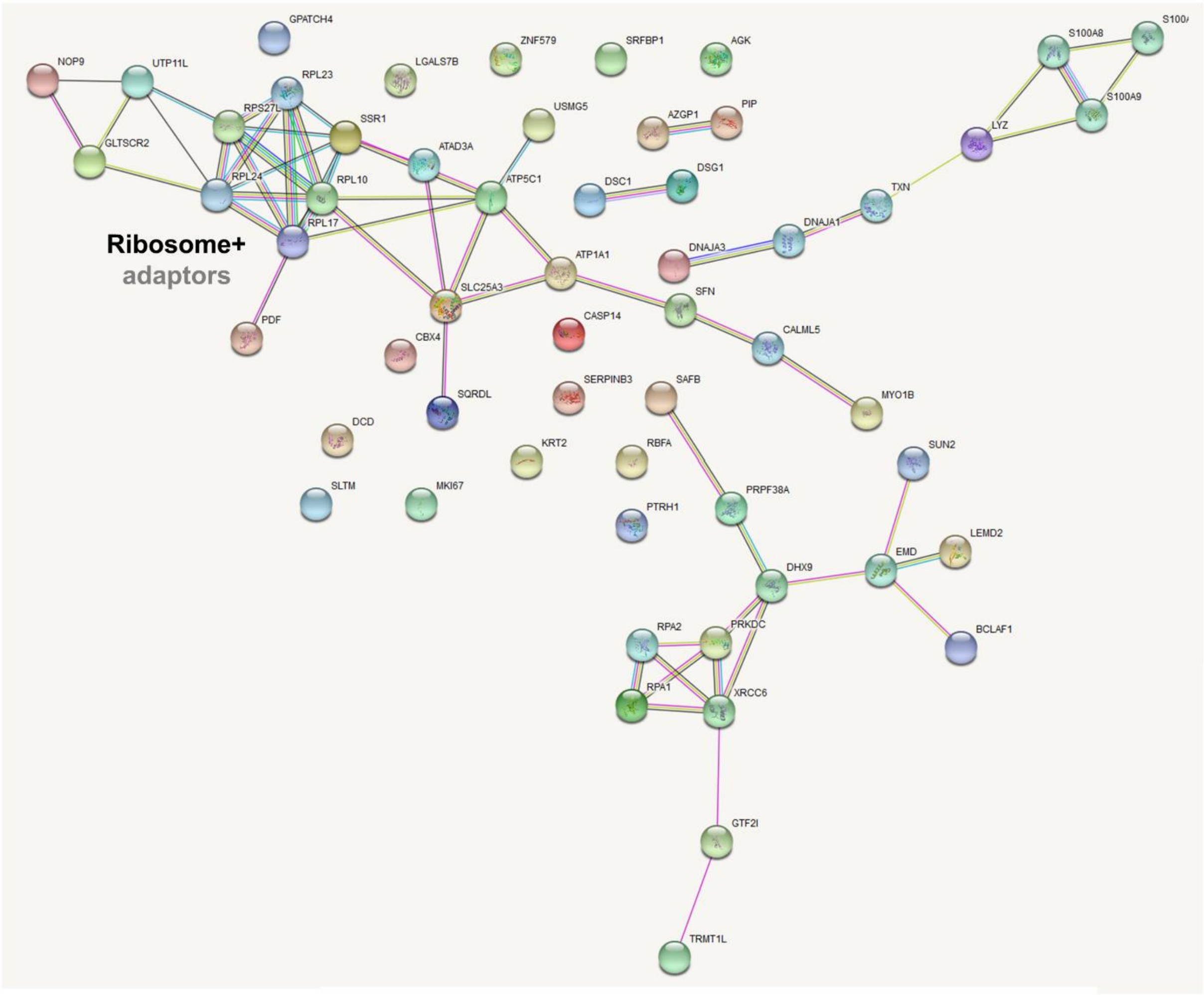


| Protein IDs | p-value | fold-change | Intensity | selective binding probability |
|---|---|---|---|---|
| P78347 | 7,94E-04 | 5,6 | 4,06E+09 | 0,97 |
| P18621 | 1,11E-03 | 1,5 | 1,65E+10 | 0,98 |
| Q9HBH1 | 1,53E-03 | 3,0 | 1,84E+08 | 1,00 |
| P43307 | 1,67E-03 | 1,7 | 4,55E+08 | 1,00 |
| P27635 | 2,17E-03 | 2,3 | 2,34E+09 | 0,94 |
| Q9UH99 | 3,01E-03 | 2,2 | 2,40E+08 | 0,92 |
| P62829 | 3,44E-03 | 1,7 | 1,60E+10 | 0,98 |
| Q96EY1 | 3,82E-03 | 1,5 | 3,90E+08 | 0,99 |
| P83731 | 4,05E-03 | 2,4 | 2,03E+09 | 0,96 |
| Q08211 | 4,12E-03 | 1,5 | 3,81E+10 | 0,99 |
| Q8NC56 | 4,75E-03 | 2,3 | 6,24E+07 | 1,00 |
| Q9NWH9 | 5,24E-03 | 2,0 | 2,41E+08 | 0,98 |
| Q9Y6N5 | 6,50E-03 | 2,8 | 1,53E+08 | 1,00 |
| Q53H12 | 6,51E-03 | 2,8 | 1,28E+08 | 0,99 |
| P78527 | 7,76E-03 | 1,8 | 6,25E+07 | -0,56 |
| Q15424 | 8,45E-03 | 2,2 | 1,07E+11 | 0,99 |
| P05023 | 1,24E-02 | 1,7 | 2,30E+08 | 0,87 |
| Q9Y3A2 | 1,90E-02 | 1,6 | 4,05E+08 | 1,00 |
| Q71UM5 | 2,51E-02 | 1,6 | 2,38E+09 | 0,98 |
| Q96IX5 | 2,64E-02 | 1,7 | 1,23E+08 | 1,00 |
| Q9NVI7 | 3,03E-02 | 1,8 | 2,00E+09 | 0,99 |
| Q86U38 | 3,73E-02 | 1,7 | 3,25E+08 | 1,00 |
| P31689 | 3,87E-02 | 1,5 | 2,21E+08 | 0,92 |
| Q5T3I0 | 5,00E-02 | 2,2 | 1,03E+08 | 0,97 |
| Q00325 | 5,27E-02 | 1,6 | 3,78E+08 | 0,94 |
| P50402 | 5,69E-02 | 1,8 | 3,28E+07 | 0,82 |
| P35908 | 5,85E-02 | 3,1 | 1,40E+10 | 0,82 |
| P31944 | 6,56E-02 | 4,5 | 1,45E+08 | 0,96 |
| Q9NZM5 | 6,62E-02 | 3,1 | 3,23E+08 | 0,99 |
| Q08554 | 6,85E-02 | 2,3 | 1,17E+08 | 0,92 |
| P15927 | 7,15E-02 | 1,9 | 1,67E+08 | 0,96 |
| P12956 | 7,73E-02 | 1,7 | 1,21E+09 | 0,96 |
| Q8NEF9 | 7,94E-02 | 1,9 | 2,42E+07 | 0,99 |
| P25311 | 8,65E-02 | 2,0 | 8,64E+07 | 0,97 |
| P10599 | 9,27E-02 | 1,7 | 4,16E+08 | 0,97 |
| O43795 | 1,01E-01 | 1,7 | 2,94E+08 | 0,90 |
| P81605 | 1,10E-01 | 2,4 | 2,93E+08 | 0,68 |
| Q8N0V3 | 1,28E-01 | 1,7 | 2,54E+07 | 1,00 |
| Q02413 | 1,30E-01 | 2,1 | 1,02E+08 | 0,71 |
| Q9NYF8 | 1,44E-01 | 1,9 | 3,51E+07 | -2,25 |
| Q8NAV1 | 1,49E-01 | 1,6 | 8,42E+07 | 0,93 |
| O00257 | 1,84E-01 | 1,5 | 5,96E+07 | 1,00 |
| Q86Y79 | 1,89E-01 | 1,9 | 2,95E+08 | 1,00 |
| P12273 | 2,06E-01 | 2,4 | 1,53E+08 | 0,96 |
| P27694 | 2,13E-01 | 1,8 | 2,76E+08 | 0,97 |
| P29508 | 2,23E-01 | 3,7 | 1,19E+08 | 0,85 |
| P05109 | 2,25E-01 | 4,6 | 5,02E+08 | 0,96 |
| Q8NAF0 | 2,29E-01 | 1,9 | 8,42E+07 | 1,00 |
| P31947 | 2,54E-01 | 2,4 | 1,37E+08 | 0,92 |
| P61626 | 2,80E-01 | 1,9 | 9,01E+07 | 0,86 |
| P31151 | 3,28E-01 | 1,9 | 7,28E+08 | 0,99 |
| Q9NZT1 | 3,47E-01 | 1,6 | 1,38E+08 | 0,96 |
| P47929 | 4,30E-01 | 3,5 | 2,55E+08 | 0,97 |
| P46013 | 4,32E-01 | 4,1 | 9,94E+08 | -0,42 |
| P06702 | 4,44E-01 | 1,9 | 2,21E+09 | 0,99 |
| Q7Z2T5 | 4,66E-01 | 2,0 | 1,50E+09 | 0,88 |
| P36542 | 5,84E-01 | 1,7 | 1,20E+08 | 0,82 |

**Figure S1**: STRING analysis of all 57 proteins from the target profiling experiment, including a table with the p-value, fold-change, intensity and selective binding probability of all proteins (FDR = 0.05, S0 = 0.1).

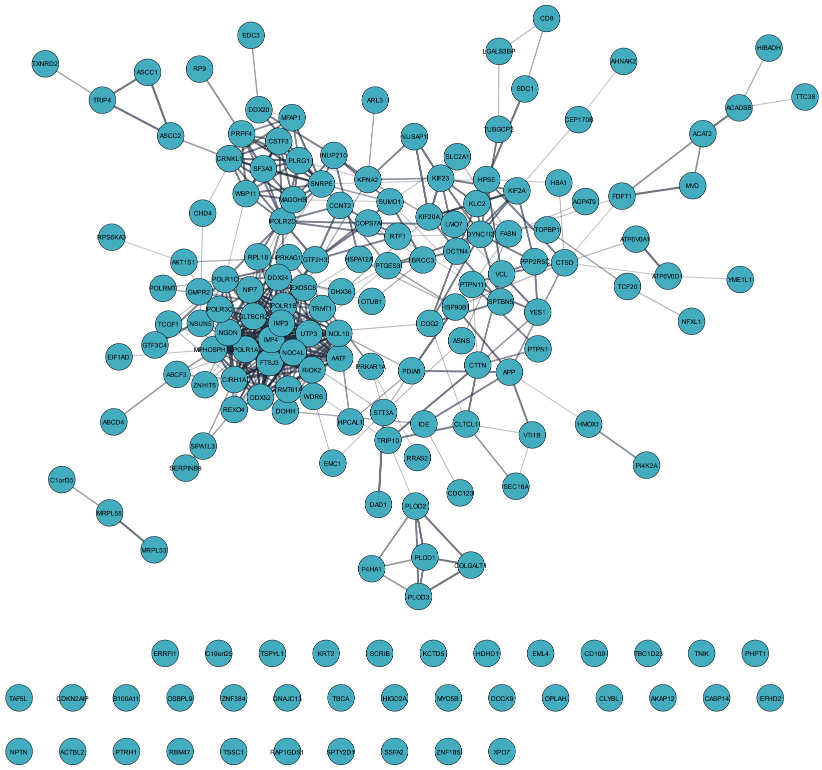


**Figure S2**: Complete STRING analysis of all significantly regulated proteins from cytoplasmic (CYT) and Nuclear extraction (NE) (FDR = 0.05, S0 = 0.1) created in Cytoscape. Several proteins from the target profiling are included in green. The confidence score was set to cutoff 0.4 with 0 additional interactors.

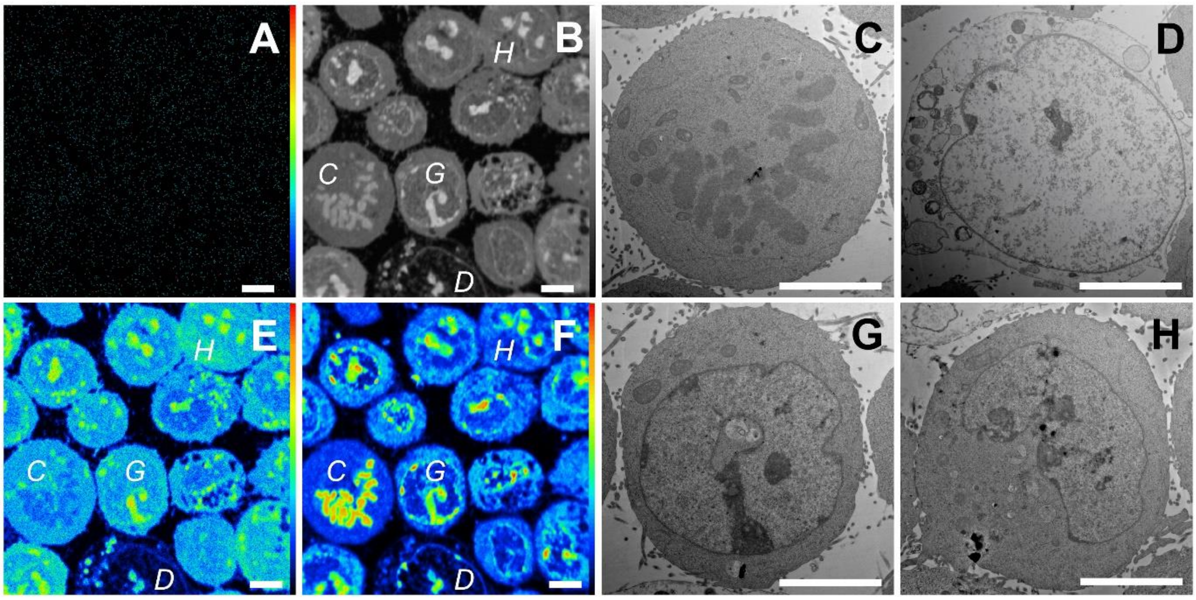


**Figure S3**. **Untreated colon carcinoma cells**. $^{102}Ru^{-}$ (A), $^{12}C^{14}N^{-}$ (B), $^{34}S^{-}$ (E) and $^{31}P^{-}$ (F) secondary ion maps (NanoSIMS) and electron micrographs (C, D, G, H) reveal the elemental distribution and general morphology of HCT116 cells (negative control). In untreated cells no signatures of severe stress induction can be observed. A cell undergoing mitosis with undisturbed endoplasmic reticulum is present (C). (D) – an example of a necrotic cell in a negative control. (G, H) are additional examples of cells with unaffected ultrastructure. Scale bars: 5 µm.

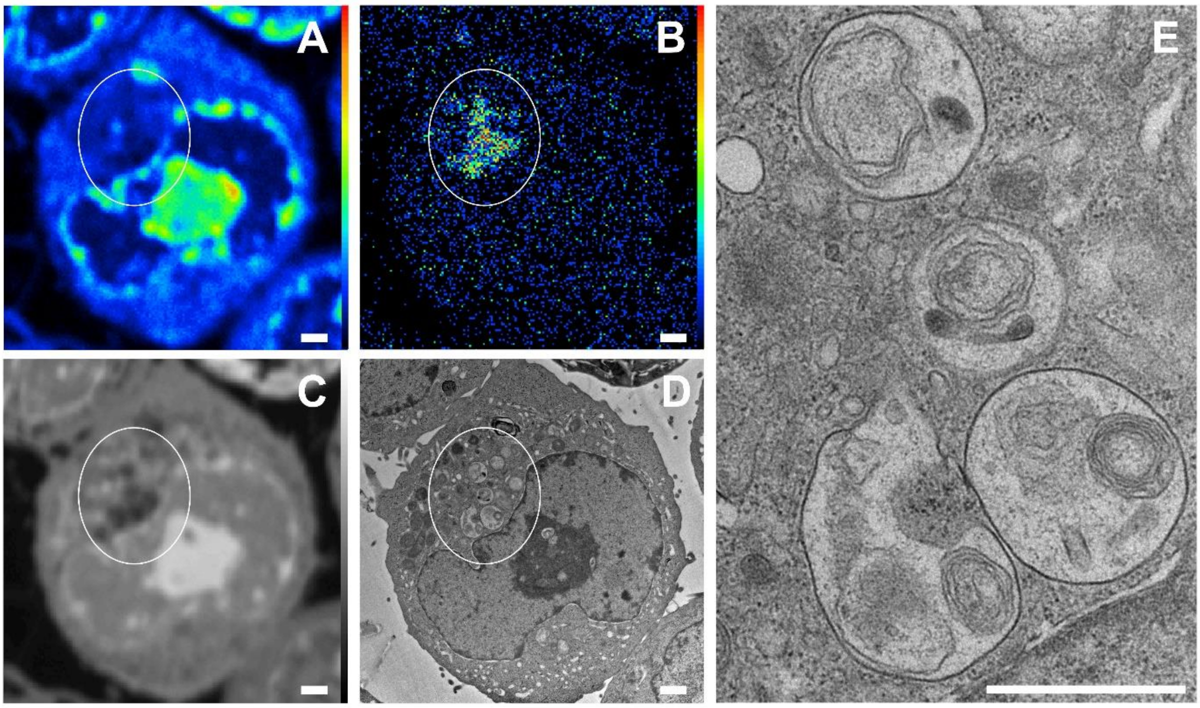


**Figure S4**. **Cytoplasmic organelles responsible for ruthenium accumulation**. Subcellular $^{102}Ru^{-}$ distribution (B) in HCT116 cells exposed to BOLD-100 (400 µM, 24 h). $^{31}P^{-}$ (A) and $^{12}C^{14}N^{-}$ (C) secondary ion maps (NanoSIMS) reveal no specific affinity of the compound to P- or N-rich molecules in the loci of $^{102}Ru^{-}$ (B) accumulation. Transmission electron micrographs (D, E) show the corresponding cellular ultrastructure. Cytoplasmic organelles that accumulate ruthenium (E, area labeled with a white oval in A-D) are featuring some major endosomal signatures, namely: rounded single membrane cytoplasmic organelles with lamellar and vesicular content. Scale bars: 1 µm,

## References


[1] P. S. Kuhn, V. Pichler, A. Roller, M. Hejl, M. A. Jakupec, W. Kandioller, B. K. Keppler, *Dalton Trans.* **2015**, *44*, 659-668.

[2] S. M. Meier, D. Kreutz, L. Winter, M. H. M. Klose, K. Cseh, T. Weiss, A. Bileck, B. Alte, J. C. Mader, S. Jana, A. Chatterjee, A. Bhattacharyya, M. Hejl, M. A. Jakupec, P. Heffeter, W. Berger, C. G. Hartinger, B. K. Keppler, G. Wiche, C. Gerner, *Angew Chem Int Ed Engl* **2017**, *56*, 8267-8271.

[3] R. L. Mayer, J. D. Schwarzmeier, M. C. Gerner, A. Bileck, J. C. Mader, S. M. Meier-Menches, S. M. Gerner, K. G. Schmetterer, T. Pukrop, A. Reichle, A. Slany, C. Gerner, *Mol. Cell. Proteomics* **2018**, *17*, 290-303.

[4] W. Huang da, B. T. Sherman, R. A. Lempicki, *Nat. Protoc.* **2009**, *4*, 44-57.

[5] D. Mellacheruvu, Z. Wright, A. L. Couzens, J. P. Lambert, N. A. St-Denis, T. Li, Y. V. Miteva, S. Hauri, M. E. Sardiu, T. Y. Low, V. A. Halim, R. D. Bagshaw, N. C. Hubner, A. Al-Hakim, A. Bouchard, D. Faubert, D. Fermin, W. H. Dunham, M. Goudreault, Z. Y. Lin, B. G. Badillo, T. Pawson, D. Durocher, B. Coulombe, R. Aebersold, G. Superti-Furga, J. Colinge, A. J. Heck, H. Choi, M. Gstaiger, S. Mohammed, I. M. Cristea, K. L. Bennett, M. P. Washburn, B. Raught, R. M. Ewing, A. C. Gingras, A. I. Nesvizhskii, *Nat. Methods* **2013**, *10*, 730-736.

[6] P. Shannon, A. Markiel, O. Ozier, N. S. Baliga, J. T. Wang, D. Ramage, N. Amin, B. Schwikowski, T. Ideker, *Genome Res* **2003**, *13*, 2498-2504.

[7] D. Szklarczyk, A. L. Gable, D. Lyon, A. Junge, S. Wyder, J. Huerta-Cepas, M. Simonovic, N. T. Doncheva, J. H. Morris, P. Bork, L. J. Jensen, C. V. Mering, *Nucleic Acids Res* **2019**, *47*, D607-D613.

[8] G. Bindea, B. Mlecnik, H. Hackl, P. Charoentong, M. Tosolini, A. Kirilovsky, W. H. Fridman, F. Pages, Z. Trajanoski, J. Galon, *Bioinformatics* **2009**, *25*, 1091-1093.

[9] V. Mathieu, C. Pirker, W. M. Schmidt, S. Spiegl-Kreinecker, D. Lotsch, P. Heffeter, B. Hegedus, M. Grusch, R. Kiss, W. Berger, *Oncotarget* **2012**, *3*, 399-413.

[10] a) A. Subramanian, P. Tamayo, V. K. Mootha, S. Mukherjee, B. L. Ebert, M. A. Gillette, A. Paulovich, S. L. Pomeroy, T. R. Golub, E. S. Lander, J. P. Mesirov, *Proc Natl Acad Sci U S A* **2005**, *102*, 15545-15550; b) V. K. Mootha, C. M. Lindgren, K. F. Eriksson, A. Subramanian, S. Sihag, J. Lehar, P. Puigserver, E. Carlsson, M. Ridderstrale, E. Laurila, N. Houstis, M. J. Daly, N. Patterson, J. P. Mesirov, T. R. Golub, P. Tamayo, B. Spiegelman, E. S. Lander, J. N. Hirschhorn, D. Altshuler, L. C. Groop, *Nat Genet* **2003**, *34*, 267-273.

[11] A. A. Legin, S. Theiner, A. Schintlmeister, S. Reipert, P. Heffeter, M. A. Jakupec, J. Mayr, H. P. Varbanov, C. R. Kowol, M. Galanski, W. Berger, M. Wagner, B. K. Keppler, *Chem. Sci.* **2016**, *7*, 3052-3061.

[12] G. Slodzian, F. Hillion, F. J. Stadermann, E. Zinner, *Appl. Surf. Sci.* **2004**, *231-232*, 874-877.
